# Collective antiskyrmion-mediated phase transition and defect-induced melting in chiral magnetic films

**DOI:** 10.1038/s41598-018-34526-0

**Published:** 2018-11-12

**Authors:** L. Pierobon, C. Moutafis, Y. Li, J. F. Löffler, M. Charilaou

**Affiliations:** 1 Laboratory of Metal Physics and Technology, Department of Materials, ETH Zurich, 8093 Zurich, Switzerland; 2 School of Computer Science, University of Manchester, M13 9PL Manchester, UK; 3 Present Address: Department of Physics, University of Louisiana at Lafayette, Lafayette, LA 70504 USA

## Abstract

Magnetic phase transitions are a manifestation of competing interactions whose behavior is critically modified by defects and becomes even more complex when topological constraints are involved. In particular, the investigation of skyrmions and skyrmion lattices offers insight into fundamental processes of topological-charge creation and annihilation upon changing the magnetic state. Nonetheless, the exact physical mechanisms behind these phase transitions remain unresolved. Here, we show numerically that it is possible to collectively reverse the polarity of a skyrmion lattice in a field-induced first-order phase transition via a transient antiskyrmion-lattice state. We thus propose a new type of phase transformation where a skyrmion lattice inverts to another one due to topological constraints. In the presence of even a single defect, the process becomes a second-order phase transition with gradual topological-charge melting. This radical change in the system’s behavior from a first-order to a second-order phase transition demonstrates that defects in real materials could prevent us from observing collective topological phenomena. We have systematically compared ultra-thin films with isotropic and anisotropic Dzyaloshinskii-Moriya interactions (DMIs), and demonstrated a nearly identical behavior for such technologically relevant interfacial systems.

## Introduction

The interplay between the symmetric Heisenberg exchange, antisymmetric Dzyaloshinskii-Moriya (DMI)^1^ and long-range magnetostatic interactions generates complex spin textures, such as helical, conical, and as shown recently^2–4^ skyrmionic phases. Skyrmions are magnetic solitons that occur on surfaces and interfaces upon rotational-symmetry breaking by either an external magnetic field^5^ or perpendicular magnetic anisotropy (PMA)^6^. The skyrmion size depends on the strength of these fields, and their handedness on the type and sign of DMI^7–9^, but regardless of these properties skyrmions always have a topological charge *Q* equal to

1 $$Q=\frac{1}{4\pi }\int \,{\bf{m}}\cdot ({{\rm{\partial }}}_{x}{\bf{m}}\times {{\rm{\partial }}}_{y}{\bf{m}})dxdy=\pm \,1,$$

with **m** the magnetization unit vector. This signifies that for both Bloch- and Néel-type skyrmions^10^ the local magnetic moment rotates by 2π from one end of a skyrmion to another, as described by a variational ansatz^11^ for a 2π domain wall. The sign of topological charge depends on the magnetization polarity of a skyrmion, but also on the direction of magnetization winding (vorticity). Objects of the opposite magnetization winding to skyrmions are called antiskyrmions^12–14^, and consequently their topological charge is opposite to that of skyrmions with the same polarity^15^.

On a surface containing more than one skyrmion, the global topological charge is the net sum resulting from all topological objects in the system. Skyrmions can be arranged in irregular clusters^16^ or rectangular lattices^17^, but mostly in hexagonal lattices, as observed for the chiral magnets MnSi^18,19^, FeCoSi^20^ and FeGe^21^. Skyrmion lattices (SkLs) are stable close to the Curie temperature in bulk systems with surfaces, and in a much wider temperature range in thin films^21,22^. In ultra-thin systems with interface-induced anisotropic DMIs, such as Ir/Co/Pt multilayers^23,24^, skyrmions form even at room temperature and in zero external magnetic field due to strong PMA. Additionally, it has been shown numerically^25^ and experimentally^26^ that a new type of magnetic solitons, namely radial vortices, also occur in the presence of weak in-plane anisotropy. Contrarily, antiskyrmion lattices have only been observed in bulk systems such as Mn–Pt–Sn^27^, and some of the current research is focused on stabilizing antiskyrmions in a wider range of materials, including thin films^28^. Nevertheless, the wide range of temperatures and materials in which skyrmionic objects exist, together with the fact that they can be controlled by relatively small current densities^29^, makes them promising candidates for future spin-based applications^30–33^.

Apart from being technologically relevant, skyrmionic spin textures offer the potential for detailed investigations into topological aspects of magnetism^34^. Specifically, since skyrmion textures are protected by their topological constraints, they cannot be continuously unwound into a trivial ferromagnetic (FM) configuration athermally without a phase transition. The exact mechanism of this kind of phase transitions, however, remains relatively unexplored so far.

In the following we demonstrate using micromagnetic simulations that skyrmion lattices undergo a magnetic field-induced phase transition where an antiskyrmion is created for each skyrmion, which results in a transient *Q* = 0 state and enables the switching of the lattice polarity. However, in the presence of even a single defect, this phase transition is replaced by a melting-type mechanism, where topological charge is gradually lost.

## Results

**Figure 1:**
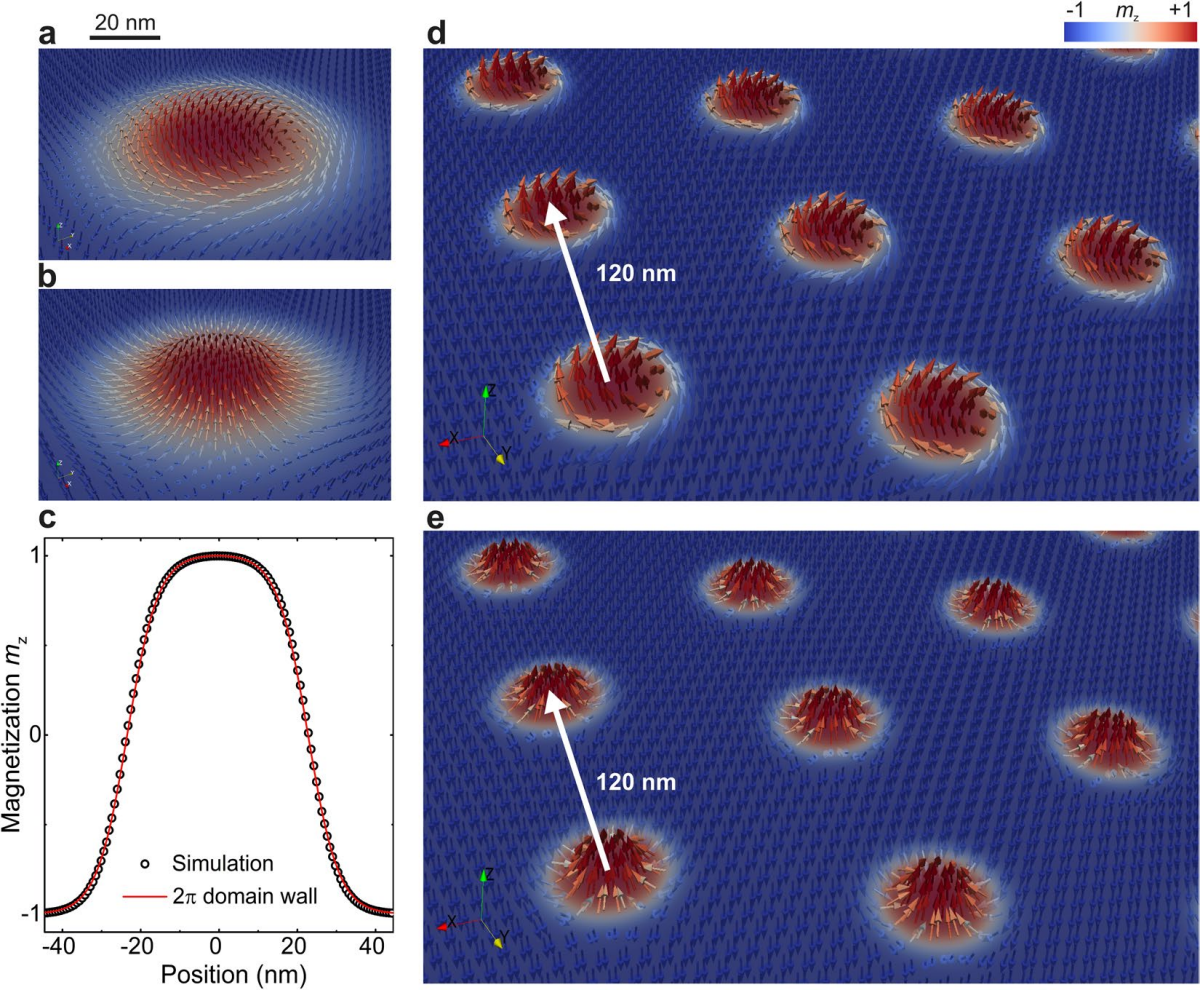
Vector plots of (**a**) Bloch and (**b**) Néel skyrmions, which are found in materials with isotropic and anisotropic DMI, respectively. (**c**) The *z*-component magnetization profiles are identical for the two kinds of skyrmions and show an excellent agreement with the magnetization profile of a 2π domain wall (solid line). (**d,e**) Skyrmions form hexagonal lattices with 6-fold symmetry, as shown in close-up vector plots of (**d**) Bloch and (**e**) Néel skyrmion lattices, where the white arrow marks the distance between neighboring skyrmions. This and all other figures in this paper, unless stated otherwise, show a system with *D* = 1.2 mJ/m^2^ and *K*_u_ = 180 kJ/m^3^.

We start by analyzing the magnetization profile of a Bloch and a Néel skyrmion in zero magnetic field in thin films with DMI and PMA. Fig. 1a–c show that the *z*-component of the magnetization for the two kinds of skyrmions is identical and can be described precisely by a variational ansatz for a 2π domain wall^11^, corresponding to a topological charge of unity, as also calculated from the relaxed spin texture. We later use this ansatz to define and calculate the radius of skyrmions by fitting it to the *z*-component of the skyrmion magnetization. In our system, skyrmions are configured in a hexagonal lattice (see Fig. 1d,e) and the global topological charge is equal to the number of skyrmions in the lattice, which in our case is *Q* = 64. Our findings are identical for bulk and interfacial DMI systems, i.e. for Bloch and Néel skyrmions. We have tested systems of different thicknesses and obtained qualitatively the same behavior (data not shown).

**Figure 2:**
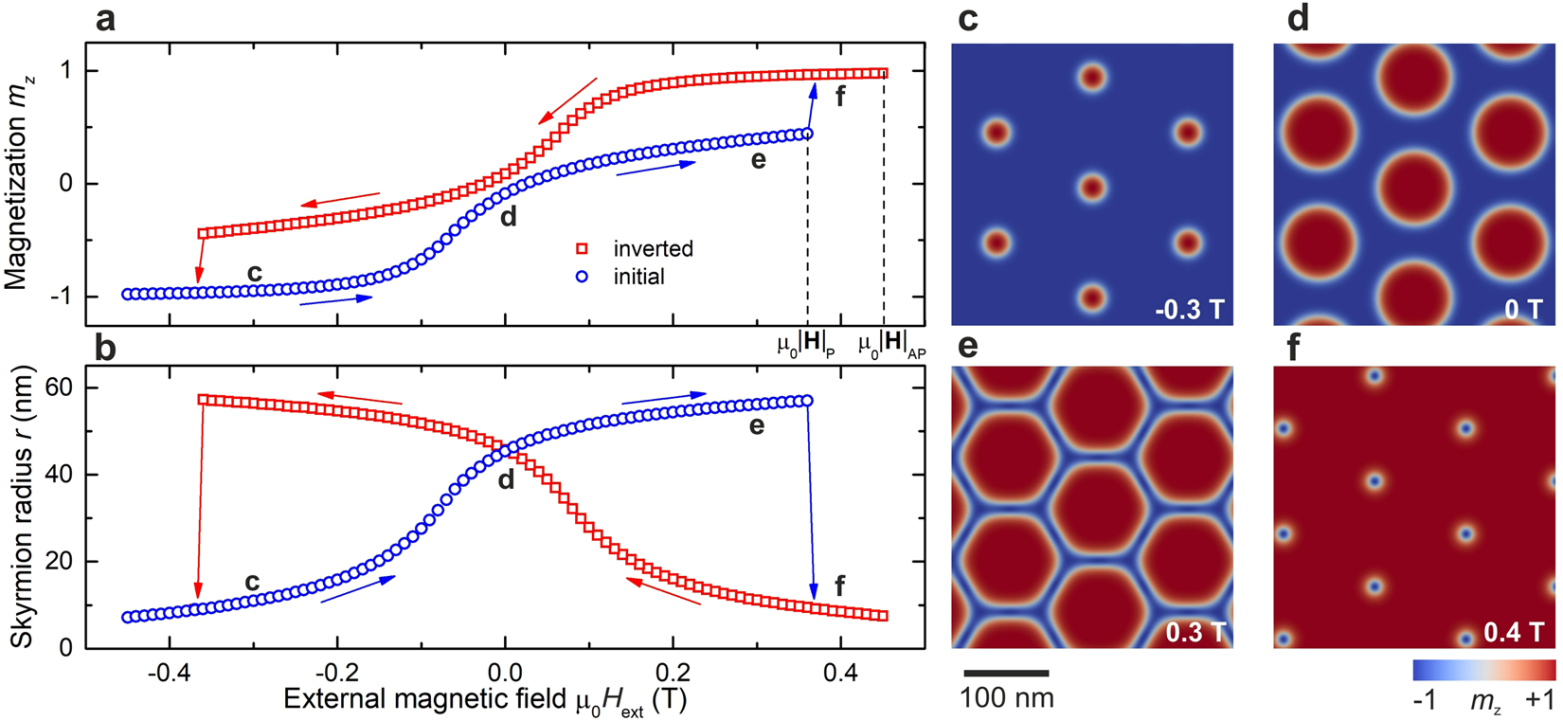
Plots of (**a**) *m*_z_ and (**b**) the radius of skyrmions as the magnetic field is swept. The inversion happens at $\pm |{{\bf{H}}}_{{\rm{P}}}|$, and the collapse to the FM state happens at $\pm |{{\bf{H}}}_{{\rm{A}}{\rm{P}}}|$. (**c**–**f**) Contour plots of the skyrmion-lattice magnetization show (**c,d**) the growth of skyrmions in parallel sweep with the topological charge localized at each skyrmion; (**e)** the hexagonal state with the topological charge delocalized on the 2π domain-wall boundary; and (**f)** the inverted skyrmion lattice. The external magnetic field is indicated in the bottom right-hand corner of the plots and the positive direction of the field is defined as that of the skyrmion-core polarization in zero field.

In order to investigate changes in skyrmion lattices as a function of external stimuli, we applied an external out-of-plane magnetic field by performing a magnetic-field sweep in the directions both parallel and antiparallel to the skyrmion-core polarization, which we define as the positive and negative directions of the external field. We observe that the skyrmions shrink and consequently the net magnetization along the *z*-axis decreases as the field is swept antiparallel to the core polarization (Fig. 2a–d, following the opposite direction of the blue arrows in Fig. 2a,b), as expected^23^. In a critical field −|**H**_AP_|, the skyrmions are annihilated and the system reaches the topologically trivial FM state through a first-order phase transition. If the field is swept parallel to the core polarization (direction of the blue arrows in Fig. 2a,b), however, the resulting behavior is much more complex. Upon increasing the field parallel to the skyrmion-core polarization, the skyrmions grow to the point where their boundaries themselves become a 2π domain wall. At this point skyrmions cannot grow anymore so they form a hexagonal state where the 2π boundaries assume the lattice symmetry, as shown in Fig. 2e. This state is special in that its topological charge distribution is different to that of the skyrmion lattices in zero field, where the skyrmions are small and well separated, and all topological charge is located on individual skyrmions. In the hexagonal state, all spin winding, and therefore all topological charge, is situated on the 2π domain-wall network, meaning that the topological charge is shared between the skyrmions, i.e., it is effectively delocalized.

From this state onwards there are two possible scenarios at a critical field $+|{{\bf{H}}}_{{\rm{P}}}|$: (i) the system undergoes a first-order phase transition from the hexagonal to the FM state; or (ii) the system undergoes another, very surprising first-order phase transition in which the skyrmion lattice inverts its magnetic polarity (Fig. 2f). This abrupt metamagnetic-like transition is characterized by a discontinuous change in the total magnetization and skyrmion radius (arrows from e to f in Fig. 2a,b). As the field is swept further, the skyrmions shrink and the inverted lattice undergoes a first-order phase transition to the FM state at $+|{{\bf{H}}}_{{\rm{AP}}}|$, in analogy to the destruction of the lattice in the antiparallel sweep.

**Figure 3:**
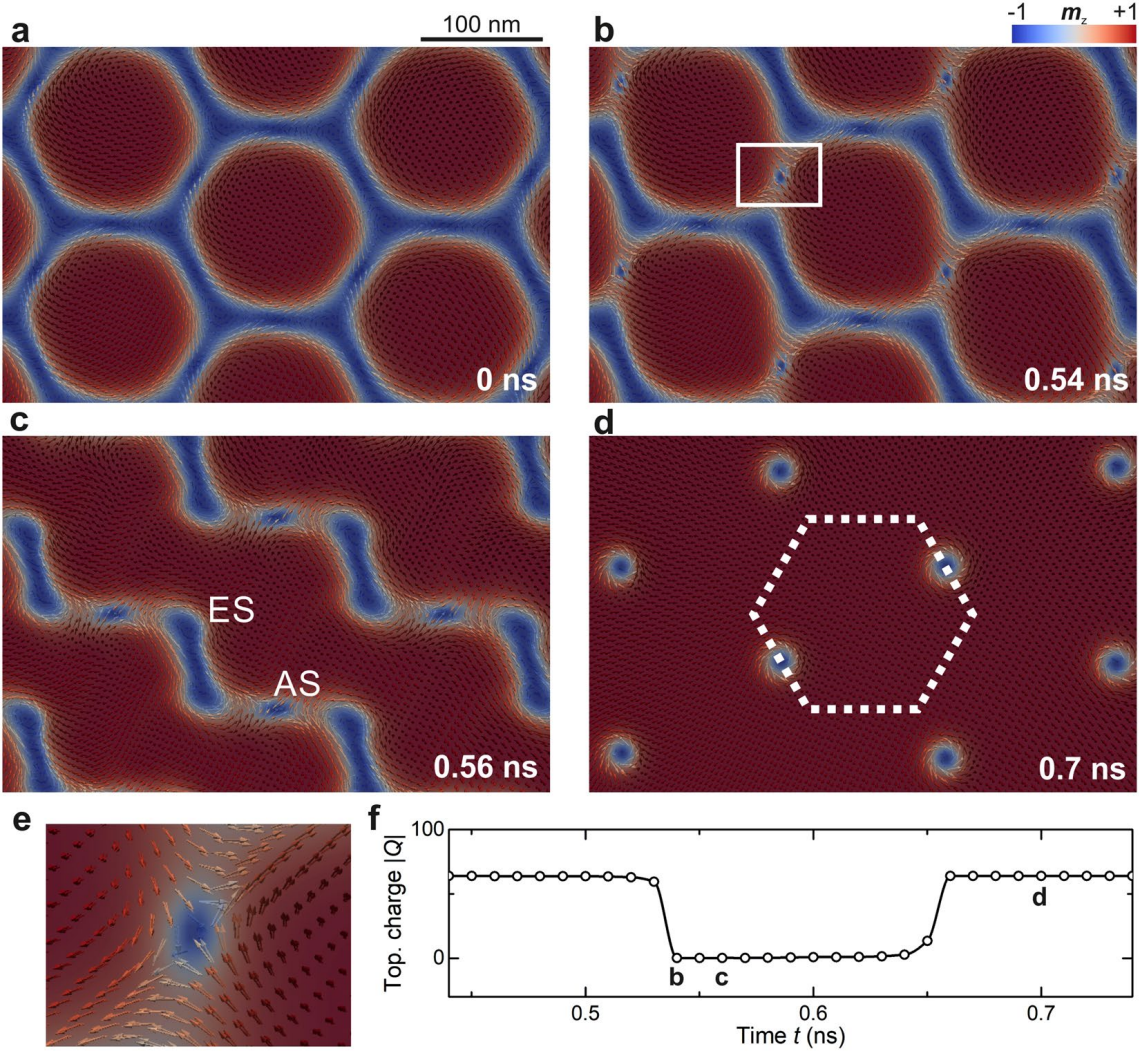
Vector plots of *m*_z_ showing the time progression of the inversion at a field slightly higher than $+|{{\bf{H}}}_{{\rm{P}}}|$ (time indicated in the bottom right-hand corner). (**a)** The hexagonal state collapses into (**b,c)** a transient state, which contains antiskyrmions and elliptical skyrmions (marked with AS and ES in **c**). (**d)** The antiskyrmions are annihilated at the end of the inversion, and the white dotted hexagon shows the position of a skyrmion before the inversion to indicate the shift of the inverted lattice with respect to the original one. (**e)** A close-up of an antiskyrmion, marked in the white rectangle in (**b)**. (**f)** The temporal evolution of the global topological charge *Q* shows that it vanishes in the transient state due to negatively charged antiskyrmions.

The metamagnetic transition of the skyrmion-lattice inversion entails striking features related to the topological charge. As illustrated in Fig. 3a,b, the inversion starts by breaking one third of the boundaries between the skyrmions. Their 2π-domain-wall shape has a topologically constraining character, which induces the creation of an antiskyrmion for each skyrmion, exactly offsetting the global topological charge. In the next step, these antiskyrmions are annihilated and new pairs of elliptical skyrmions and antiskyrmions are created from the remaining boundaries (Fig. 3c), keeping the global topological charge at zero. At the end of the inversion, all antiskyrmions become annihilated, which restores the global topological charge to its original value. Here, new cores of inverted skyrmions form from the elliptical skyrmions, which are situated at the vertices of the skyrmions in the original SkL (Fig. 3d), i.e., the inverted skyrmions have emerged from the 2π boundaries (see Supplementary Video 1).

**Figure 4:**
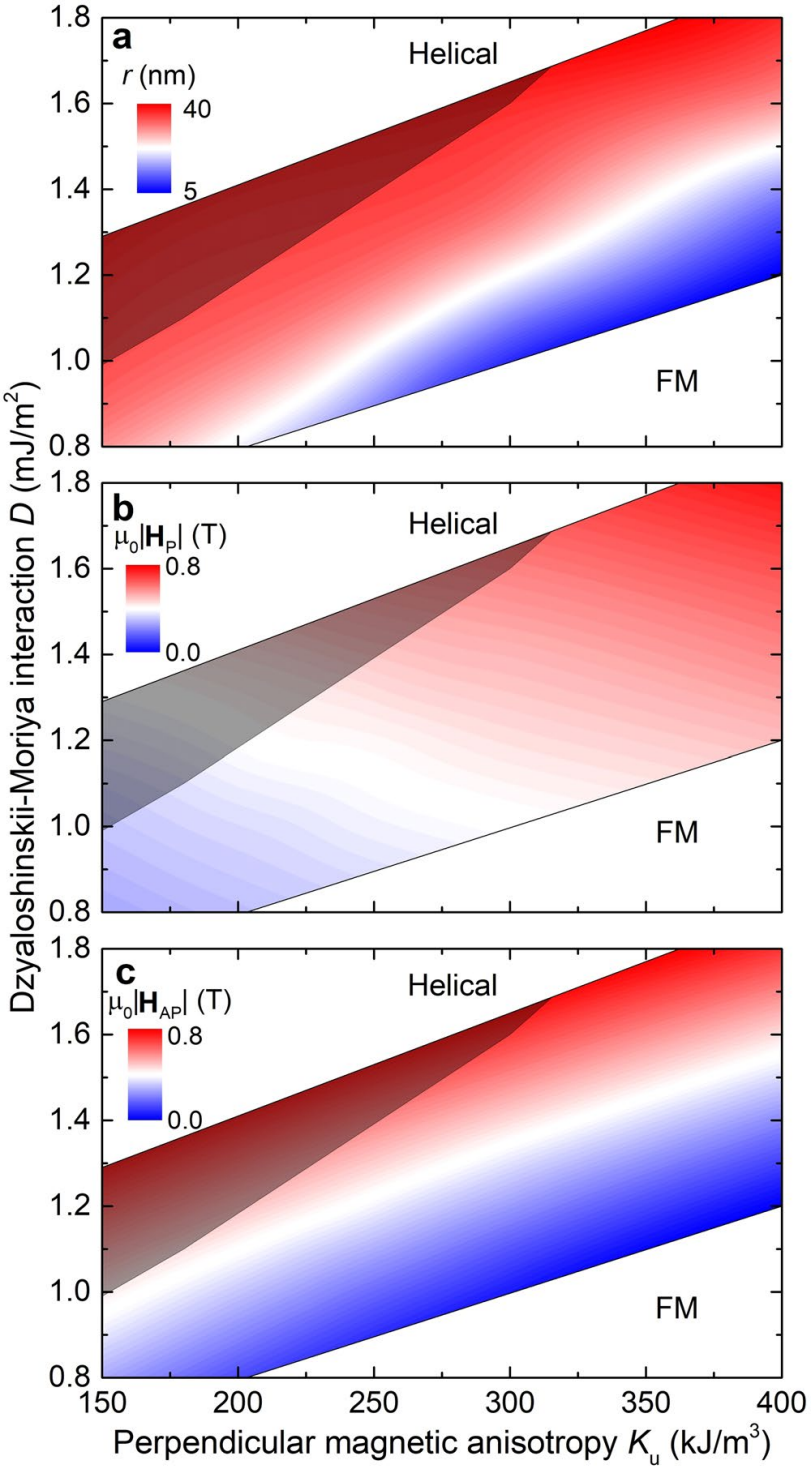
Contour plots showing the range of PMA and DMI in which skyrmion lattices (colored) are stable in zero magnetic field. The helical phase in the diagram is stable above the skyrmion lattice phase, and the ferromagnetic phase below. (**a)** The radius of skyrmions *r* increases in materials with strong DMI and low PMA; (**b)** $|{{\bf{H}}}_{{\rm{P}}}|$ increases with both PMA and DMI; and (**c)** $|{{\bf{H}}}_{{\rm{A}}{\rm{P}}}|$ increases with DMI but decreases with PMA. This means that the inversion happens only in the materials with low PMA (represented by the shaded areas in the diagrams), where $|{{\bf{H}}}_{{\rm{P}}}| < {\bf{H}} < |{{\bf{H}}}_{{\rm{AP}}}|$.

The phenomena described above depend strongly on the intrinsic material parameters. For a DMI strength between 1 and 2 mJ/m^2^, comparable to the material values of intrinsically chiral magnets or Co/Pt-based multilayers, we have investigated the range of PMA (i) for which skyrmion lattices are stable in zero magnetic field; and (ii) for which the inversion happens. We have compared bulk and interfacial systems, i.e., systems with isotropic and anisotropic DMIs respectively, and found a nearly identical behavior. The SkL phase is stable in the range of *K*_u_ = 150–800 kJ/m^3^ and the skyrmions grow with decreasing PMA and increasing DMI (Fig. 4a), in agreement with literature^7–9^. The inversion can only occur if the inverted SkL is stable in the field range $|{{\bf{H}}}_{{\rm{P}}}| < {\bf{H}} < |{{\bf{H}}}_{{\rm{AP}}}|$. The values of $|{{\bf{H}}}_{{\rm{P}}}|$ and $|{{\bf{H}}}_{{\rm{AP}}}|$, and therefore whether or not the SkL inversion occurs, depend on the material properties. As shown in Fig. 4b,c, $|{{\bf{H}}}_{{\rm{P}}}|$ increases with increasing PMA, but $|{{\bf{H}}}_{{\rm{AP}}}|$ decreases with increasing PMA, so that the criterion limits the occurrence of inversion to materials with low PMA (shaded area in Fig. 4).

This behavior can be explained by a competition of the various interactions in the system. PMA acts to decrease the size of the skyrmions in order to minimize the area of the spins misaligned with the easy axis. This is in competition with the Zeeman energy, which acts to increase the size of the skyrmions in order to align them with the external magnetic field. At low PMA, the skyrmion lattices reach the hexagonal state in a relatively small magnetic field. Due to the confined skyrmion winding in the hexagonal lattice, the skyrmions cannot transition into the trivial ferromagnetic state, so they invert their polarization in order to align more area with the magnetic field. At high PMA, the skyrmion lattices are relatively small in zero magnetic field, and a large magnetic field is required for them to reach the hexagonal state. In such high magnetic fields, the DMI energy is small compared to the Zeeman energy, so that the ferromagnetic state is more favorable. Supplementary Fig. 1 shows a further analysis of the energetics for various PMA and DMI.

The findings described above assume an infinite ideal system. However, real materials, either bulk or thin films, contain defects that may strongly affect the magnetic state, particularly in multilayers with interfacial DMIs. It is therefore important to study the effect of defects on the SkL phase and its stability, which is essential for enabling the functionality of skyrmion-based devices^30–33^.

**Figure 5:**
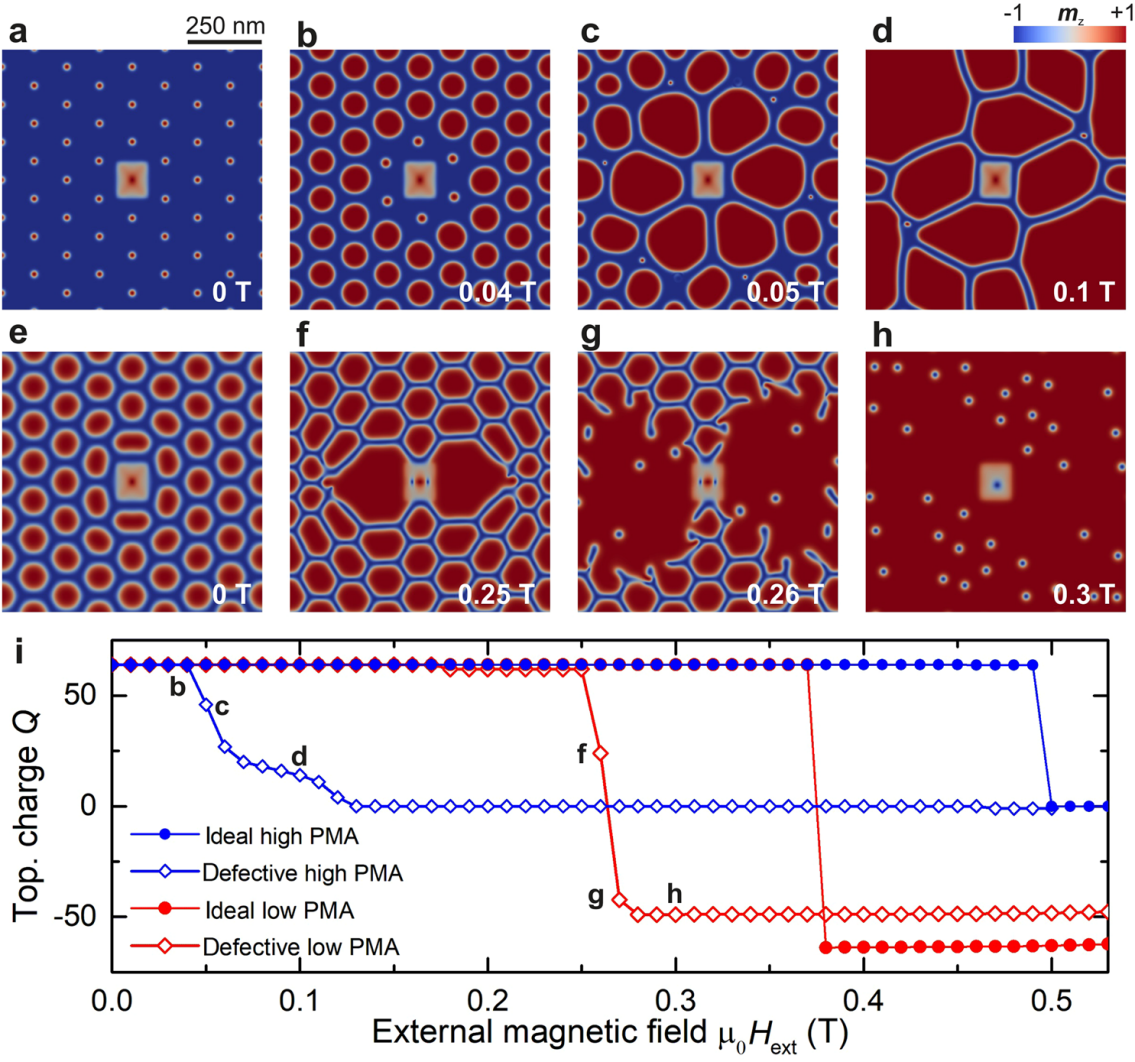
Contour plots of *m*_z_ showing defect-induced melting due to a defect with zero PMA in the region of a single skyrmion for a material with (**a**–**d**) PMA = 350 kJ/m^3^, which shows no inversion in the ideal case, and (**e**–**h**) PMA = 180 kJ/m^3^, exhibiting inversion in the ideal case. The value of the external magnetic field in each plot is marked in the bottom right-hand corner. (**i**) Comparison between the ideal and defective lattices by a plot of *Q* against the external field. Note that defect-induced melting does not happen if the magnetic field is swept antiparallel to the skyrmion-core polarization; instead, the skyrmions shrink until the FM state is reached just like in the ideal system.

In our simulations, we have implemented single and multiple defects of three different kinds: (i) a local variation of DMI or PMA, (ii) a local distortion in the skyrmion lattice, and (iii) a vacancy in the skyrmion lattice. We find that while defects do not significantly affect the system’s behavior when the field is swept antiparallel to the skyrmion-core polarization, they compromise the stability of the SkL phase and dramatically modify the associated magnetization processes when the field is swept parallel. In fact, the annihilation of the lattice starts at the defect site, because the skyrmion of the same shape and size as in the rest of the lattice is not stable within the region of the defect. Thus, due to the high degree of confinement in the system, the skyrmions start twisting, deforming, and inhomogeneously growing at the defect site. This starts an avalanche-like effect which results in elliptical instabilities^35^ and consequently in a gradual loss of topological charge. At high PMA (Fig. 5a–d,i), where the inversion does not occur even in ideal systems, all topological charge is lost. This is in contrast to low PMA, where inversion occurs in ideal systems. Here, not all topological charge is destroyed for defective systems because some skyrmions still undergo inversion (Fig. 5e–i). Figure 5i demonstrates that the stability of skyrmion lattices is less compromised by defects at low PMA than at high PMA. This, together with our finding that inversion still occurs at low PMA regardless of having a single defect or multiple defects, suggests that the inversion may be experimentally observable even for defective systems (see Supplementary Videos 2 and 3).

Importantly, the critical field in which the lattice is destroyed is strongly reduced in defective systems (see Fig. 5i), and the transition changes from first-order to second-order (see Supplementary Fig. 2), where we observe gradual lattice-melting behavior. This destabilization of skyrmion lattices due to the presence of defects illustrates the importance of symmetry. In an ideal SkL, the application of a parallel field leads to a hexagonal state, where the skyrmions are stabilized by a shared 2π domain-wall network, i.e., the skyrmion lattice is protected by the topology of both the skyrmions themselves and the 2π boundaries. This suggests that the topological charge of an ideal lattice is *delocalized* when the constraint is enforced by the 2π domain-wall network. In contrast, a system with even a single defect destabilizes the lattice due to the loss of topological protection provided by the boundaries of the skyrmion at the defect site. Since the latter acts as a topological-charge sink, the density of defects crucially determines the skyrmion-lattice stability. We have also investigated the effect of edges in the system by removing periodic boundary conditions, and our findings show that they act similar to defects, i.e., the unwinding of spin textures at edges leads to defect-induced melting. This further signifies the importance of confinement for skyrmion-lattice systems, where the stability of the whole system depends strongly on its symmetry and regularity.

Finally, in order to gain more insight into the energetics of the system, we have calculated the total energy for the inverted skyrmion-lattice state shown in Figs 2 and 3 and for a trivial ferromagnetic state in the same magnetic field. Our findings indicate that the inverted skyrmion lattice is in a local energy minimum and that its energy is only 1 μeV per atom higher than that of the trivial ferromagnetic state. This is relatively small compared to the energy scales of up to 500 μeV per atom for the exchange and Zeeman energies. Thus, we can conclude that thermal fluctuations would not compromise the experimental realization of inversion.

**Figure 6:**
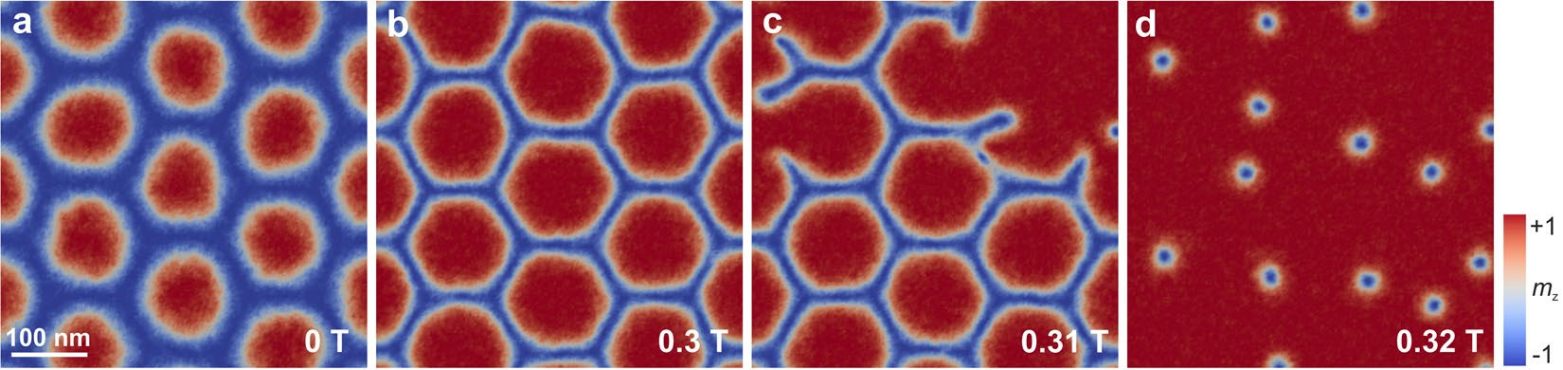
Contour plots of *m*_z_ in a system with an internal fluctuating magnetic field, mimicking thermal effects at *T* = 100 K, and a superimposed external field, which is indicated in the right bottom corner. (**a**) Thermal fluctuations induce irregularities in zero field, but this does not prevent the system from reaching (**b**) the hexagonal state. (**c**) Via defect-induced melting, (**d**) some skyrmions invert their polarization, as illustrated in Fig. 5.

To confirm this, we have investigated finite-temperature effects on the system by applying a randomly fluctuating thermal magnetic field to simulate thermal agitation. As shown in Fig. 6, random irregularities, which form due to thermal fluctuations, effectively behave like defects, i.e spin unwinding at irregularities results in defect-induced melting. This means that finite-temperature effects reduce the skyrmion-lattice stability, but do not prevent skyrmions from inverting their polarization. These results confirm that the state in the system is not simply subject to the energetics, but strongly depends on the topological constraints. In conclusion, the topological constraints are much stronger in an ideal material hosting a perfect skyrmion lattice than in a defect-containing material, where the constraints are weaker and the magnetization unwinds at the defect. The latter destabilizes the skyrmion lattice both at zero and non-zero temperatures.

## Discussion

Our study reveals the complex underlying mechanisms of topological-charge creation and annihilation in thin magnetic films with perpendicular magnetic anisotropy, and both isotropic (bulk) and anisotropic (interfacial) Dzyaloshinskii-Moriya interaction. We have found that upon the application of an external field to ideal infinite films the skyrmion-lattice phase undergoes a first-order phase transition, either to a topologically trivial ferromagnetic state or to an inverted skyrmion-lattice phase via the transient formation of antiskyrmions. The first-order character of both phase transitions in the ideal lattice is due to a delocalization of the topological charge within a 2π domain-wall network and the consequent collective response to an external field. In the presence of even a single defect, however, the skyrmion-lattice phase becomes unstable and collapses gradually through a defect-induced melting process, where the defect site acts as a topological-charge sink. These findings emphasize the importance of imperfections in materials and their implications on the stability of topologically non-trivial spin textures, and demonstrate that the consideration of defects is paramount for the analysis of experimental data. This provides a basis for a much wider scope of experiments on skyrmion lattices, particularly concerning the development of materials for skyrmion-based devices.

## Methods

We have performed high-resolution micromagnetic simulations to investigate in detail the phase transition of skyrmion lattices. We have studied ultrathin films where the skyrmion-lattice phase is stable at temperatures low enough for our micromagnetic simulations to be valid, as in most cases we did not consider finite-temperature effects.

The total energy density *F* consists of: (i) ferromagnetic exchange; (ii) perpendicular magnetic anisotropy; (iii) isotropic (bulk) or anisotropic (interfacial) Dzyaloshinskii-Moriya interaction; (iv) Zeeman coupling to an external magnetic field; and (v) dipole-dipole interactions:

2 $$F=A{({\rm{\nabla }}\cdot {\bf{m}})}^{2}+D{\bf{m}}\cdot ({\rm{\nabla }}\times {\bf{m}})-{K}_{{\rm{u}}}{({m}_{{\rm{z}}})}^{2}-{\mu }_{0}{M}_{{\rm{s}}}{\bf{m}}\cdot {{\bf{H}}}_{{\rm{e}}{\rm{x}}{\rm{t}}}-\frac{{\mu }_{0}{M}_{{\rm{s}}}}{2}{\bf{m}}\cdot {{\bf{H}}}_{{\rm{d}}{\rm{e}}{\rm{m}}{\rm{a}}{\rm{g}}},$$

where **m **= **M**/*M*_s_ is the magnetization unit vector with **M** the magnetization and *M*_*s*_ the saturation magnetization, *A* is the exchange stiffness, *D* is the strength of the DMI (either bulk or interfacial), *K*_u_ is the first-order uniaxial anisotropy constant, **H**_ext_ is the external magnetic field, and **H**_demag_ is the local demagnetizing field due to dipole-dipole interactions. The *z*-component of the magnetization is perpendicular to the film plane.

We computed the magnetic state by solving the Landau–Lifshitz–Gilbert (LLG) equation of motion:

3 $${\partial }_{t}{\bf{m}}=-\,\gamma ({\bf{m}}\times {{\bf{H}}}_{{\rm{eff}}})+\alpha ({\bf{m}}\times {\partial }_{t}{\bf{m}}),$$

where *γ* is the electron gyromagnetic ratio, *α* is the dimensionless damping parameter, and ${{\bf{H}}}_{{\rm{e}}{\rm{f}}{\rm{f}}}=-\,{{\rm{\partial }}}_{{\bf{m}}}F/{\mu }_{0}{M}_{{\rm{s}}}$ is the effective magnetic field in the material consisting of external and internal magnetic fields, which depend on the material parameters. The LLG simulations have been done with mumax3^36^, a finite-difference GPU-based program.

For both isotropic and anisotropic DMI systems we have considered a wide range of material parameters that are valid for many real materials^23,37–40^: *D* = 1.0–2.0 mJ/m^2^ and *K*_u_ = 20–800 kJ/m^3^. The values of $A=8.78$ pJ/m and ${M}_{{\rm{s}}}=385$ kA/m were also taken from real materials, e.g. FeGe^37,40^, and the damping parameter was defined as $\alpha =0.1$. While *A* and *M*_s_ were fixed throughout the study, the DMI and PMA strength were varied to obtain more detailed insight into the physics of skyrmion lattices and the related effects of energetics.

The thin films were discretized in a 480 × 480 × 2 mesh (sample dimensions 832 nm × 960 nm × 4 nm) with periodic boundary conditions imposed on the operators such as the exchange interactions in *x*- and *y*-directions, which are reflected by the magnetization of the sample. Additionally, different cell sizes (always less than half the exchange length^11^ ${\delta }_{{\rm{ex}}}=\sqrt{2{A}_{{\rm{ex}}}/{\mu }_{0}{M}_{{\rm{s}}}^{2}}\approx 10$ nm) were used to verify the numerical stability of the simulations.

The external magnetic field was always applied and swept perpendicular to the film plane, and the quantities recorded at each field step were: **m**, **M**, *Q*, the total energy density of the system and the individual contributions to it.

Finite-temperature effects were added by aplying a randomly-fluctuating magnetic field **H**_them_ defined as:

4 $${{\bf{H}}}_{{\rm{t}}{\rm{h}}{\rm{e}}{\rm{r}}{\rm{m}}}={\boldsymbol{\eta }}({\rm{s}}{\rm{t}}{\rm{e}}{\rm{p}})\sqrt{\frac{2\alpha {k}_{{\rm{B}}}T}{{B}_{{\rm{s}}{\rm{a}}{\rm{t}}}{\mu }_{0}\gamma Vt}},$$

where **η** is a random vector from a standard normal distribution whose value is changed with every time step, *k*_B_ the Boltzmann constant, *T* the temperature, *B*_sat_ the magnetic induction, *V* the cell volume and *t* = 10^−13^ s the time step.

## Electronic supplementary material

SI Guide

Video 1

Video 2

Video 3

## Data Availability

All datasets generated and/or analyzed in this study are available from the corresponding authors on request. They are not made publicly available online due to their memory size.

## References

[1] Moriya T (1960). Anisotropic superexchange interaction and weak ferromagnetism. Physical Review.

[2] Rössler UK, Bogdanov AN, Pfleiderer C (2006). Spontaneous skyrmion ground states in magnetic metals. Nature.

[3] Bode M (2007). Chiral magnetic order at surfaces driven by inversion asymmetry. Nature.

[4] Nagao M (2013). Direct observation and dynamics of spontaneous skyrmion-like magnetic domains in a ferromagnet. Nature Nano..

[5] Buhrandt, S. & Fritz, L. Skyrmion lattice phase in three-dimensional chiral magnets from monte carlo simulations. *Physical Review B* **88** (2013).

[6] Wilson, M. N., Butenko, A. B., Bogdanov, A. N. & Monchesky, T. L. Chiral skyrmions in cubic helimagnet films: The role of uniaxial anisotropy. *Physical Review B* **89** (2014).

[7] Ezawa, M. Giant skyrmions stabilized by dipole-dipole interactions in thin ferromagnetic films. *Physical Review Letters* **105** (2010). 10.1103/PhysRevLett.105.197202 21231193

[8] Guslienko KY (2015). Skyrmion state stability in magnetic nanodots with perpendicular anisotropy. IEEE Magnetics Letters.

[9] Rohart S, Thiaville A (2013). Skyrmion confinement in ultrathin film nanostructures in the presence of dzyaloshinskii-moriya interaction. Phys. Rev. B.

[10] Hellman F (2017). Interface-induced phenomena in magnetism. Rev. Mod. Phys..

[11] Braun H-B (2012). Topological effects in nanomagnetism: from superparamagnetism to chiral quantum solitons. Advances in Physics.

[12] Zhang, S., Petford-Long, A. K. & Phatak, C. Creation of artificial skyrmions and antiskyrmions by anisotropy engineering. *Scientific Reports* **6** (2016). 10.1038/srep31248 PMC4978955 27507196

[13] Stier M, Häusler W, Posske T, Gurski G, Thorwart M (2017). Skyrmion–anti-skyrmion pair creation by in-plane currents. Phys. Rev. Lett..

[14] Hoffmann, M. *et al*. Antiskyrmions stabilized at interfaces by anisotropic dzyaloshinskii-moriya interactions. *Nature Communications* **8** (2017). 10.1038/s41467-017-00313-0 PMC5566362 28827700

[15] Koshibae W, Nagaosa N (2016). Theory of antiskyrmions in magnets. Nature Communications.

[16] Li W (2016). Emergence of skyrmions from rich parent phases in the molybdenum nitrides. Physical Review B.

[17] Heinze S (2011). Spontaneous atomic-scale magnetic skyrmion lattice in two dimensions. Nature Physics.

[18] Muhlbauer S (2009). Skyrmion lattice in a chiral magnet. Science.

[19] Tonomura A (2012). Real-space observation of skyrmion lattice in helimagnet MnSi thin samples. Nano Letters.

[20] Yu XZ (2010). Real-space observation of a two-dimensional skyrmion crystal. Nature.

[21] Yu XZ (2010). Near room-temperature formation of a skyrmion crystal in thin-films of the helimagnet FeGe. Nature Materials.

[22] Huang SX, Chien CL (2012). Extended skyrmion phase in EpitaxialFeGe(111)thin films. Physical Review Letters.

[23] Moreau-Luchaire C (2016). Additive interfacial chiral interaction in multilayers for stabilization of small individual skyrmions at room temperature. Nature Nanotechnology.

[24] Boulle O (2016). Room-temperature chiral magnetic skyrmions in ultrathin magnetic nanostructures. Nature Nanotechnology.

[25] Siracusano G (2016). Magnetic radial vortex stabilization and efficient manipulation driven by the dzyaloshinskii-moriya interaction and spin-transfer torque. Physical Review Letters.

[26] Karakas V (2018). Observation of magnetic radial vortex nucleation in a multilayer stack with tunable anisotropy. Scientific Reports.

[27] Nayak AK (2017). Magnetic antiskyrmions above room temperature in tetragonal heusler materials. Nature.

[28] Camosi L, Rougemaille N, Fruchart O, Vogel J, Rohart S (2018). Micromagnetics of antiskyrmions in ultrathin films. Physical Review B.

[29] Jonietz F (2010). Spin transfer torques in MnSi at ultralow current densities. Science.

[30] Parkin SSP, Hayashi M, Thomas L (2008). Magnetic domain-wall racetrack memory. Science.

[31] Sampaio J, Cros V, Rohart S, Thiaville A, Fert A (2013). Nucleation, stability and current-induced motion of isolated magnetic skyrmions in nanostructures. Nature Nanotechnology.

[32] Fert A, Cros V, Sampaio J (2013). Skyrmions on the track. Nature Nanotechnology.

[33] Fert A, Reyren N, Cros V (2017). Magnetic skyrmions: advances in physics and potential applications. Nature Reviews Materials.

[34] Nakajima T (2017). Skyrmion lattice structural transition in mnsi. Science Adv..

[35] Moutafis C, Komineas S, Bland JAC (2009). Dynamics and switching processes for magnetic bubbles in nanoelements. Physical Review B.

[36] Vansteenkiste A (2014). The design and verification of MuMax3. AIP Advances.

[37] Beg M (2015). Ground state search, hysteretic behaviour, and reversal mechanism of skyrmionic textures in confined helimagnetic nanostructures. Scientific Reports.

[38] Yang H, Boulle O, Cros V, Fert A, Chshiev M (2016). Controlling dzyaloshinskii-moriya interaction via chirality dependent layer stacking, insulator capping and electric field. Scientific Reports.

[39] Ericsson T, Karner W, Haeggstroem L, Chandra K (1981). Magnetic structure of cubic FeGe. Physica Scripta.

[40] Yamada H (2003). Electronic structure and magnetism of FeGe with b20-type structure. Physica B: Condensed Matter.

