# Supplementary material for: Collective antiskyrmion-mediated phase transition and defect-induced melting in chiral magnetic films
Source: Sci Rep. 2018 Nov 12;8:16675. doi: 10.1038/s41598-018-34526-0 (PMC6232090; doi:10.1038/s41598-018-34526-0)
Supplement: Supplementary file 1 — SI Guide [file 41598_2018_34526_MOESM1_ESM.pdf]

Collective antiskyrmion-mediated phase transition and defect-induced melting in chiral magnetic films

L. Pierobon[1], C. Moutafis[2], Y. Li[2], J. F. Löffler[1], M. Charilaou[1,3]

[1]*Laboratory of Metal Physics and Technology, Department of Materials, ETH Zurich, 8093 Zurich, Switzerland*

[2]*School of Computer Science, University of Manchester, M13 9PL Manchester, UK*

[3]*Present address: Department of Physics, University of Louisiana at Lafayette, Lafayette, LA 70504, USA*

SUPPLEMENTARY NOTES

We have investigated the effect of skyrmion separation on their size and found that at low PMA and strong DMI the skyrmions grow linearly with their separation. An energy-density analysis shows there is a minimum in the exchange-energy density at a skyrmion separation of 50 – 60 nm, which is associated to their growth (Fig. 1). The separation at which the minimum exists is larger in materials with lower PMA and stronger DMI. Contrarily, the minimum does not exist at high PMA and weak DMI, where the growth of skyrmions is inhibited after they reach a radius of about 15 nm.

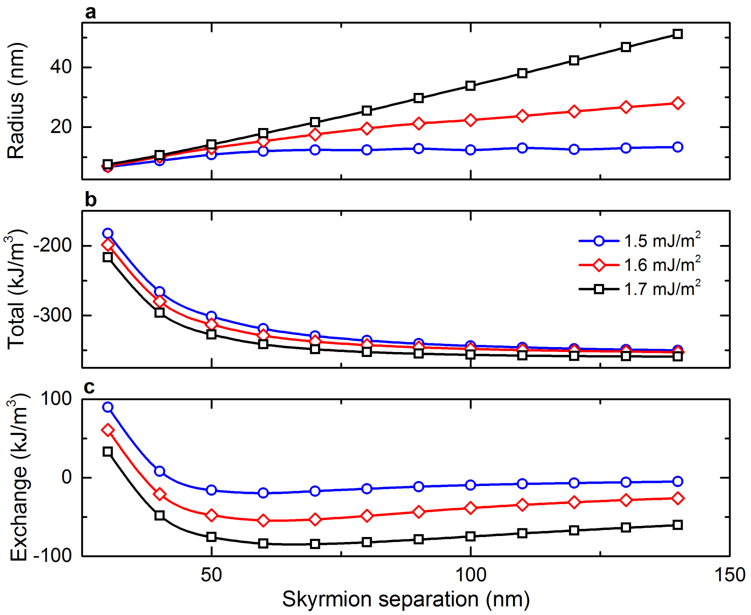

Suppl. Fig. 1: Plots of (**a**) skyrmion radius, (**b**) total energy density, and (**c**) exchange energy density against skyrmion separation in the lattice for three different values of DMI strength. At strong DMI and low PMA (the latter result not shown in the graph), the skyrmion radius grows with skyrmion separation. This might be promoted by the existence of an exchange energy minimum, whose position is at around 50 – 60 nm and increases for stronger DMI (black and red lines) and lower PMA. The growth is inhibited at low DMI and high PMA, probably because the minimum in exchange energy vanishes (blue line).

To determine the transition order of defect-induced melting, we have analyzed how the susceptibility (i.e., the second derivative of energy with respect to the external field) varies with the external field. Supplementary Figure 2 shows that the susceptibility reveals peaks in the critical field when defect-induced melting starts, and this discontinuity demonstrates that defect-induced melting is a second-order phase transition.

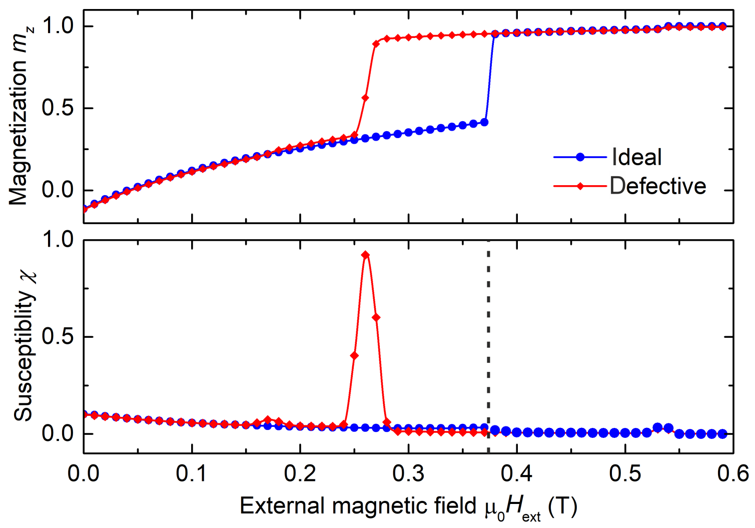

Suppl. Fig. 2: Plots of (**a**) magnetization (the first derivative of energy with respect to the external field) and (**b**) susceptibility (the second derivative) versus the external magnetic field for the ideal system (shown in Figs. 2 and 3 in the main text) and for the defective system (with low PMA; see Figs. 5e-i in the main text). The magnetization is clearly discontinuous and the susceptibility reaches infinity (indicated by the dotted line) during the inversion in the ideal case, which is characteristic of a first-order phase transition. In constrast, the magnetization is continuous for defect-induced melting with the susceptibility being discountinuous, characteristic of a second-order phase transition.

SUPPLEMENTARY VIDEOS

Video 1: Skyrmion-lattice inversion in a slightly supercritical field involves the breaking of 2π domain-wall boundaries and a consequent formation of antiskyrmions that exactly offset the topological charge. In the next step, these antiskyrmions are destroyed and new pairs of elliptical

skyrmions and antiskyrmions emerge from the remaining boundaries, exhibiting rotation-like motion. These antiskyrmions finally vanish at the end of the inversion, and the topological charge is fully restored.

Video 2: Skyrmion-lattice defect-induced melting at high PMA as the external magnetic field is ramped up from 0 to 0.2 T. The skyrmions start growing irregularly at the defect sites, which results in eliptical instabilites, the destruction of individual skyrmions, and a complete loss of topological charge at a much lower critical field.

Video 3: Skyrmion-lattice defect-induced melting at low PMA as the external magnetic field is ramped up from 0 to 0.5 T. Similarly as in Video 2, the skyrmions grow and become destroyed at the defect site, but a few skyrmions survive the inversion so that a finite topological charge is restored.
